# Prognostic Value of the Distribution of Lymph Node Metastasis in Locally Advanced Rectal Cancer After Neoadjuvant Chemoradiotherapy

**DOI:** 10.3389/fsurg.2021.749575

**Published:** 2021-11-17

**Authors:** Bin Chen, Xing Liu, Yiyi Zhang, Jinfu Zhuang, Yong Peng, Ye Wang, Yong Wu, Shoufeng Li, Yuanfeng Yang, Guoxian Guan

**Affiliations:** ^1^Department of Colorectal Surgery, The First Affiliated Hospital of Fujian Medical University, Fuzhou, China; ^2^Department of Colorectal Surgery, Fujian Medical University Union Hospital, Fuzhou, China

**Keywords:** lymph node, metastasis, rectal cancer, neoadjuvant chemoradiotherapy, prognosis

## Abstract

**Background:** The objective of this study is to assess the prognostic value of lymph node metastasis distribution (LND) in locally advanced rectal cancer (LARC) after neoadjuvant chemoradiotherapy (nCRT).

**Methods:** This study included 179 patients with pathological stage III LARC who underwent nCRT followed by radical surgery. LND was classified into three groups: LND1, lymph node metastasis at the mesorectum (140/179, 78.2%); LND2, lymph node metastasis along the inferior mesenteric artery trunk nodes (26/179, 14.5%); LND3, lymph node metastasis at the origin of the IMA (13/179, 7.3%). Clinicopathologic characteristics were analyzed to identify independent prognostic factors.

**Result:** LND showed better stratification for 3-year DFS (LND1 66.8, LND2 50, and LND3 15.4%, *P* < 0.01) compared to the ypN (3-year DFS: N1 59.9 and N2 60.3%, *P* = 0.34) and ypTNM (3-year DFS: IIIA 68.6%, IIIB 57.5%, and IIIC 53.5, *P* = 0.19) staging systems. Similar results were found for 3-year LRFS and DMFS. According to multivariate survival analysis, LND was shown to be an independent prognostic factor for DFS, LRFS, and DMFS in patients with positive lymph nodes (*P* < 0.01, in all cases).

**Conclusion:** LND is an independent prognostic factor in stage III rectal cancer after nCRT. LND can be used as a supplementary indicator for the ypTNM staging system in patients with LARC after nCRT.

## Introduction

Neoadjuvant chemoradiotherapy (nCRT) followed by total mesorectal excision (TME) is widely used as a multimodal treatment for rectal cancer. This approach has achieved good results with respect to tumor downstaging, reducing local recurrence, and improving R0 resection (1–3). The latest guidelines recommend nCRT followed by radical surgery as the standard treatment for patients with locally advanced rectal cancer (LARC) (4, 5). Currently, the ypTNM staging system is the most common indicator for evaluating the prognosis of patients with LARC after nCRT. However, it has certain limitations in the assessment of prognosis due to the influence of radiotherapy (6, 7). Particularly, radiotherapy changes the prognostic value of the ypN staging system by affecting the number and status of lymph nodes (8, 9). Therefore, a new lymph node classification method is needed to accurately assess the prognosis of rectal cancer after nCRT.

The distribution of lymph node metastasis (LND) has been proved to be an important prognostic factor for colorectal cancer, which remains controversial (10–14). Although the scope of radiotherapy is below the sacral promontory level, it still causes considerable effect on the lymph nodes in the mesorectum and those around the inferior mesenteric artery (IMA) (15, 16). Moreover, there is individual heterogeneity in the response to nCRT. The prognostic significance of LND in rectal cancer may change due to the influence of nCRT. Till date, only few studies have reported the prognostic impact of LND after nCRT in patients with rectal cancer (16–18). Therefore, this study uses the lymph node classification system according to the Japanese Classification of Colorectal Carcinoma (19) to analyze the prognostic value of LND in rectal cancer after nCRT.

## Methods

### Patients

This study retrospectively analyzed patients with LARC who underwent nCRT combined with TME surgery at the Colorectal Surgery Department of the Fujian Union Hospital from December 2010 to December 2016. The inclusion criteria were as follows: histologically proven rectal cancer, clinical stage before treatment was T3-T4 or N positive stage; and tumor located within 10 cm from the anal verge. The following patients were excluded: those with distant metastasis before surgery, those undergoing emergency surgery or palliative resection, and those undergoing local excision or wait-and-see treatment. Our institutional review board approved this study. All patients provided written informed consent.

### Treatment

Evaluation and staging strategies before and after nCRT include digital rectal examination, analysis of serum carcinoembryonic antigen (CEA) and serum carbohydrate antigen 19-9, chest X-ray or computed tomography (CT), pelvic magnetic resonance imaging (MRI), and transrectal ultrasound. The preoperative long-course radiotherapy protocol comprised 50.4 Gy radiation delivered as five fractions of 1.8 Gy per week for 5 consecutive weeks followed by an additional dose of 5.4 Gy. Preoperative chemotherapy was initiated on the first day of radiotherapy, and it included two different regimens: fluorouracil plus oxaliplatin (FOLFOX/CapeOX) and fluorouracil only.

Surgery was performed 6–12 weeks after the radiation therapy was completed. Surgical techniques for rectal cancer, such as TME and IMA high ligation, have been standardized in our institution. Starting ~3–4 weeks after surgery, patients received adjuvant chemotherapy for 6 months. Two different chemotherapy regimens were used: FOLFOX and CapeOX.

### Pathologic Examination and Definitions

Lymph nodes were separated individually from the adipose connective tissue of the specimen before formalin fixation by the surgeons. According to the Japanese Classification of Colorectal Carcinoma 19, we classified LND into three groups: LND1, lymph node metastasis at the mesorectum (perirectal lymph nodes); LND2, lymph node metastasis along the inferior mesenteric artery trunk nodes (intermediate lymph nodes); and LND3, lymph node metastasis at the origin of the IMA (main lymph nodes). In addition, referring to study by Lee et al. (16), we categorized LND in a different manner: LNDm, lymph node metastasis at the mesorectum; LNDp, metastasis at the proximal lymph nodes (intermediate lymph nodes or main lymph nodes); and LNDmp, metastasis at both perirectal and proximal lymph nodes. The specimens were examined by at least two experienced pathologists using a standard method. When <12 lymph nodes were found, the specimen was re-examination to guarantee the maximum lymph node yield.

The location of the rectum (above or below the peritoneal reflection) was determined by preoperative abdominopelvic MRI and intraoperative findings. Final pathologic features were restaged according to the seventh edition of the American Joint Committee on Cancer TNM staging system, at the time of data review. The largest tumor diameter determined *via* pathologic examination was defined as the tumor size. The circumferential resection margin was considered positive when the margin was <1 mm. The Tumor Regression Grade (TRG) was established by implementing the AJCC criteria as follows: TRG0—complete response with no visible tumor cells remaining; TRG1—moderate response with a single or small group of tumor cells remaining; TRG2—minimal response with the residual cancer outgrown by fibrosis; and TRG3—poor response with extensive residual cancer with minimal or no tumor death (20).

### Follow-Up and Endpoint

A follow-up evaluation was performed every 3 months for the first 2 years, then every 6 months for the next 3 years, and annually thereafter. At each visit, a physical examination, CEA determination, chest X-ray or CT scans, and abdominopelvic MRI or CT scans were performed. A colonoscopy was performed annually after surgery. A positron emission tomography (PET) examination was conducted when needed. The endpoints in this study were disease-free survival (DFS), local recurrence-free survival (LRFS) and distant recurrence-free survival (DMFS) after surgery. Local recurrence was defined as recurrence in the pelvis cavity confirmed by histopathology or imaging. Distant metastasis was defined as recurrence outside the pelvis. We collected data by a survey of original medical records and access to the hospital information system.

### Statistical Analysis

Statistical analysis was performed using SPSS version 25.0 (SPSS INC., Chicago). Categorical variables were expressed as numbers with percentages and were compared using a chi-square test or Fisher's exact test, as appropriate. Continuous variables were described as means ± standard deviations and analyzed using Kruskal-Wallis test. Survival rates were estimated and compared using the Kaplan-Meier method and log-rank test. The Cox proportional hazard model was used for univariate analysis and multivariate survival analysis. Results were considered significant at *P* < 0.05, and significant variables in univariate analysis were used in the multivariate analysis.

## Results

### Clinical and Pathological Characteristics

A total of 179 patients with stage III LARC were included. Our study group comprised 114 (63.7%) men and 65 (36.3%) women. The average age was 53.5 ± 12.1 years. The interval between nCRT and surgery was 8.7 ± 1.4 weeks. The average distance of the tumor from the anal verge was 6.2 ± 2.5 cm. The average number of lymph nodes harvested was 15.5 ± 7.0 and the average number of positive lymph nodes was 2.8 ± 4.1. LND1, LND2, and LND3 were identified in 140 (78.2%), 26 (14.5%), and 13 (7.3%) patients.

The clinicopathological factors of the patients according to their LND status are shown in Table 1. The preoperative serum CEA level, ypN stage, and ypTNM stage were significantly greater in patients with a higher LND status than those with LND1. Open surgery, complications, perineural invasion, lymphovascular invasion, and CRM involvement were more frequent in patients with a higher LND status than those with LND1.

**Table 1:** Clinicopathological factors according to the distribution of lymph node metastasis (LND).

**Factors**	**LND1**	**LND2**	**LND3**	***p*-value**
	***n* = 140**	***n* =26**	***n* = 13**	
**Gender**				
Male	88 (62.9)	16 (61.5)	10 (76.9)	0.64
Female	52 (37.1)	10 (38.5)	3 (23.1)	
Age (year)	54.2 ± 12.2	52.7 ± 11.9	48.4 ± 11.2	0.24
**ASA class**				
1	111 (79.3)	22 (84.6)	12 (92.3)	0.69
2	28 (20.0)	4 (15.4)	1 (7.7)	
3	1 (0.7)	0 (0.0)	0 (0.0)	
Interval to surgery (weeks)	8.8 ± 1.2	8.9 ± 1.6	7.6 ± 2.5	0.19
**Preoperative chemotherapy regimen**				
Fluorouracil only	95 (67.9)	14 (53.8)	5 (38.5)	0.06
Fluorouracil + oxaliplatin	45 (32.1)	12 (46.2)	8 (61.5)	
**Distance from anal verge (cm)**				
≤ 5	58 (41.4)	13 (50.0)	5 (38.5)	0.76
>5	82 (58.6)	13 (50.0)	8 (61.5)	
**Preoperative CEA (ng/mL)**				
<5	122 (89.7)	22 (84.6)	8 (61.5)	0.046
≥5	14 (10.3)	4 (15.4)	5 (38.5)	
Unknown	4 (2.9)	0 (0.0)	0 (0.0)	
**Preoperative CA199 (ng/mL)**				
<37	130 (92.9)	24 (92.3)	11 (84.6)	0.67
≥37	8 (5.7)	2 (7.7)	2 (15.4)	
Unknown	2 (1.4)	0 (0.0)	0 (0.0)	
**Operative method**				
Laparoscopic	106 (75.7)	16 (61.5)	5 (38.5)	0.01
Open	34 (24.3)	10 (38.5)	8 (61.5)	
**Type of surgery**				
Anterior resection	118 (84.3)	18 (69.2)	11 (84.8)	0.18
APR + Hartmann's	22 (15.7)	8 (30.8)	2 (15.4)	
Operation time (minutes)	221.5 ± 63.1	222.0 ± 64.0	221 ± 115.38	0.10
Blood loss (ml)	94.0 ± 112.4	93.5 ± 94.3	115.4 ± 89.3	0.37
**Complication**				
No	117 (83.6)	15 (57.7)	12 (92.3)	<0.01
Yes	23 (16.4)	11 (43.2)	1 (7.7)	
**Tumor size (cm)**				
<2	39 (27.9)	5 (19.2)	1 (7.7)	0.26
≥2	101 (72.1)	21 (80.8)	12 (92.3)	
**ypT stage**				
0–2	41 (29.3)	9 (34.6)	3 (23.1)	0.75
3–4	99 (70.7)	17 (65.4)	10 (76.9)	
**ypN stage**				
1	123 (87.9)	18 (69.2)	4 (30.8)	<0.01
2	17 (12.1)	8 (30.8)	9 (69.2)	
**ypTNM stage**				
IIIA	36 (25.7)	7 (26.9)	2 (15.4)	<0.01
IIIB	96 (68.6)	18 (69.2)	5 (38.5)	
IIIC	8 (5.7)	6 (3.8)	6 (46.2)	
No. of lymph nodes harvested	15.2 ± 6.2	14.7 ± 8.1	19.8 ± 11.0	0.17
**Tumor differentiation**				
Well moderately differentiated	112 (80.0)	22 (84.6)	10 (76.9)	0.79
Poorly differentiated, others^a^	28 (20.0)	4 (15.4)	3 (23.1)	
**Tumor regression grade**				
0	5 (3.6)	2 (7.7)	0 (0.0)	0.07
1	45 (32.1)	5 (19.2)	3 (23.1)	
2	76 (54.3)	16 (61.5)	5 (38.5)	
3	14 (10.0)	3 (11.5)	5 (38.5)	
**Perineural invasion**				
Negative	128 (91.4)	21 (80.8)	8 (61.5)	<0.01
Positive	12 (8.6)	5 (19.2)	5 (38.5)	
**Lymphovascular invasion**				
Negative	134 (95.7)	25 (96.2)	10 (76.9)	0.036
Positive	6 (4.3)	1 (3.8)	3 (23.1)	
**CRM involvement**				
Negative	139 (99.3)	26 (100.0)	11 (84.6)	0.021
Positive	1 (0.7)	0 (0.0)	2 (15.4)	

*ASA, American Society of Anesthesiologists; CEA, serum carcinoembryonic antigen; CA199, carbohydrate antigen 19-9; CRM, circumferential resection margin; ^a^Included mucinous and signet ring cell carcinoma; APR, abdominoperineal resection*.

### Univariate and Multivariate Survival Analysis

With a median follow-up time of 39 months (range 1–95 months), 73 (40.7%) patients experienced recurrence, 61 (30.1%) exhibited distant metastasis, and 21 (11.7%) exhibited local recurrence; 9 (5.0%) patients were diagnosed with both distant metastasis and local recurrence. The 3-year disease-free survival (DFS), local recurrence-free survival rates (LRFS) and distant metastasis-free survival (DMFS) of all patients were 60, 71.2, and 61.7%, respectively.

**Figure 1:**
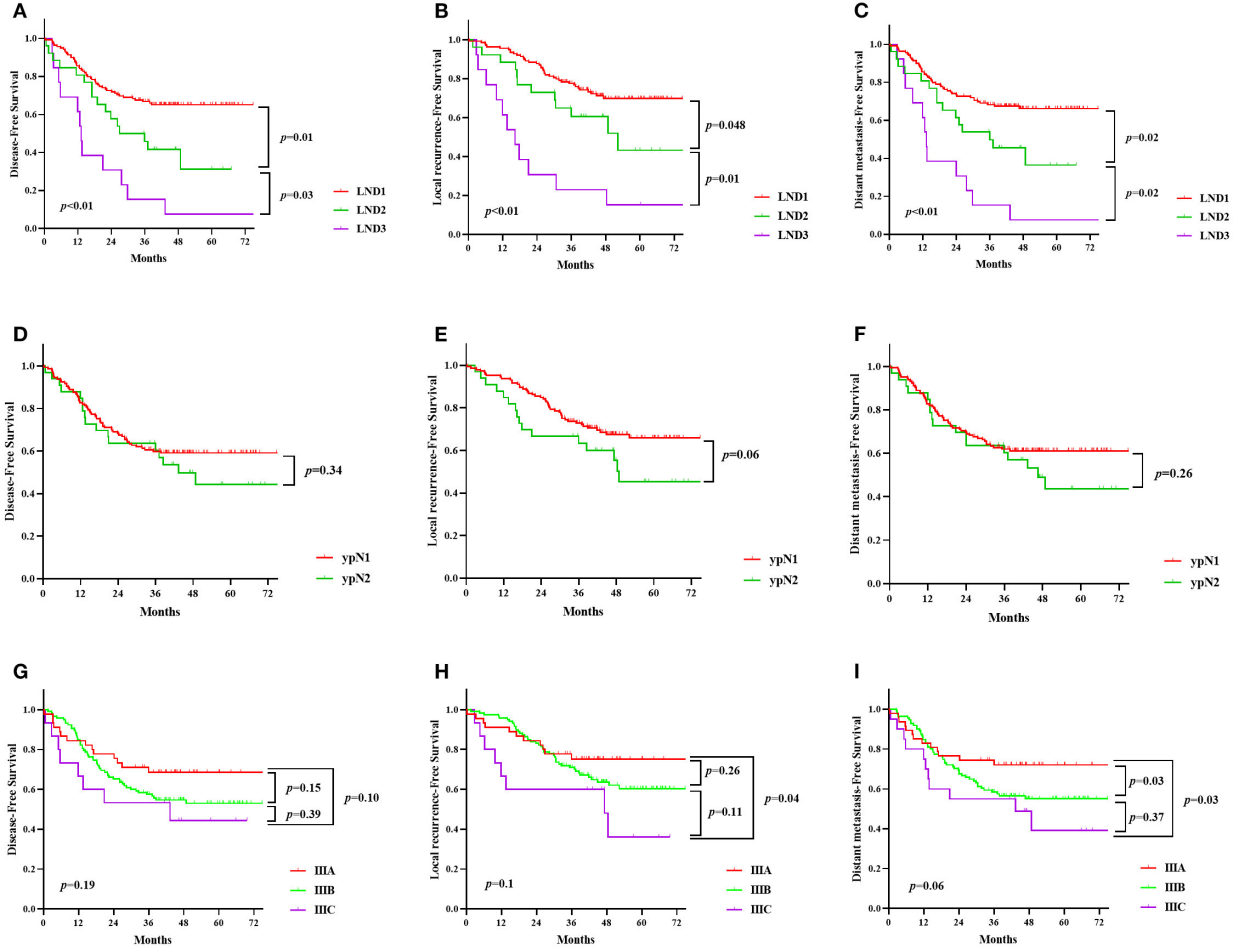
Kaplan-Meier survival curve according to lymph node distribution (LND) **(A–C)**, the ypN stage **(D–F)** and ypTNM stage **(G–I)**. **(A,D,G)**, disease-free survival; **(B,E,H)**, local recurrence-free survival; **(C,F,I)**, distant metastasis-free survival.

The comparison between the survival curves is displayed in Figure 1. The disease-free survival curves among the LND groups significantly differed, according to the results of the log-rank test (*P* < 0.01); the 3-year DFS of the patients with LND1, LND2, and LND3 were 66.8, 50, and 15.4%, respectively. However, no significant difference in the 3-year DFS was observed with respect to the ypN and ypTNM stages (*P* = 0.342 and 0.191, respectively). Similar results were observed for 3-year LRFS and DMFS. The results of univariate and multivariate survival analysis for DFS, LRFS, and DMFS are shown in Tables 2–4. LND was shown to be an independent prognostic factor for DFS, LRFS, and DMFS (all *P* < 0.01).

**Table 2:** Cox regression analysis of factors for 3-year disease-free survival in ypN+ rectal cancer patients after nCRT.

**Factors**	**Univariate analysis**			**Cox regression analysis**		
	**HR**	**95%CI**	***P***	**HR**	**95%CI**	***P***
Gender (male vs. female)	1.018	0.639–1.623	0.94			
Age (year)	0.993	0.975–1.012	0.48			
**ASA class**						
1	Reference		0.77			
2	1.012	0.566–1.809	0.97			
3	2.064	0.286–14.913	0.47			
Interval to surgery (weeks)	0.870	0761–0.994	0.04	0.967	0.844–1.108	0.63
Preoperative chemotherapy regimen (fluorouracil + oxaliplatin vs. fluorouracil only)	1.324	0.840–2.086	0.23			
Distance from anal verge (>5 vs. ≤ 5 cm)	0.770	0.490–1.208	0.26			
Preoperative CEA (≥5 vs. <5 ng/mL)	2.298	1.319–4.002	<0.01	1.442	0.784–2.652	0.24
Preoperative CA199 (≥37 vs. <37 ng/mL)	1.001	0.981–1.021	0.93			
Operation method (open vs. laparoscopic)	1.597	1.002–2.546	0.049	1.112	0.653–1.895	0.70
Type of surgery (APR + Hartmann's vs. anterior resection)	1.422	0.819–2.469	0.21			
Operation time (minutes)	1.000	0.997–1.004	0.87			
Blood loss (ml)	1.001	0.999–1.003	0.39			
Complication	1.367	0.813–2.299	0.24			
Tumor size (≥2 vs. <2 cm)	1.563	0.888–2.750	0.12			
ypT stage (ypT3-4 vs. ypT0-2)	1.740	1.002–3.022	0.049	1.763	1.012–3.070	0.045
ypN stage (ypN2 vs. ypN1)	1.298	0.757–2.227	0.34			
ypTNM stage			0.20			
IIIA	Reference					
IIIB	1.534	0.852–2.762	0.15			
IIIC	2.116	0.888–5.046	0.09			
No. of lymph nodes harvested	1.004	0.968–1.041	0.83			
LND			<0.01			<0.01
LND1	Reference			Reference		
LND2	2.041	1.158–3.596	0.014	2.152	1.219–3.799	<0.01
LND3	4.587	2.422–8.686	<0.01	4.465	2.356–8.463	<0.01
Tumor differentiation (poorly differentiated, others^a^ vs. Well moderately differentiated)	1.284	0.748–2.202	0.37			
Tumor regression grade			0.34			
0	Reference					
1	1.374	0.321–5.879	0.67			
2	1.616	0.391–6.681	0.51			
3	2.481	0.560–10.998	0.23			
Perineural invasion	1.704	0.919–3.159	0.09			
Lymphovascular invasion	1.741	0.703–4.314	0.23			
CRM involvement	2.805	0.686–11.463	0.15			

*CEA, serum carcinoembryonic antigen; CA199, carbohydrate antigen 19-9; APR, abdominoperineal resection; LND, lymph node distribution; ^a^Included mucinous and signet ring cell carcinoma; CRM, circumferential resection margin*.

**Table 3:** Cox regression analysis of factors for 3-year local recurrence-free survival in ypN+ rectal cancer patients after nCRT.

**Factors**	**Univariate analysis**			**Cox regression analysis**		
	**HR**	**95%CI**	***P***	**HR**	**95%CI**	***P***
Gender (male vs. female)	0.744	0.433–1.277	0.28			
Age (year)	0.987	0.966–1.008	0.22			
**ASA class**						
1	Reference		0.52			
2	0.946	0.492–1.818	0.87			
3	3.119	0.429–22.684	0.26			
Interval to surgery (weeks)	0.825	0.721–0.944	<0.01	0.9	0.772–1.050	0.18
Preoperative chemotherapy regimen (fluorouracil +oxaliplatin vs. fluorouracil only)	1.662	1.006–2.744	0.047	0.899	0.768–1.051	0.18
Distance from anal verge (>5 vs. ≤ 5cm)	0.760	0.462–1.251	0.28			
Preoperative CEA (≥5 vs. <5 ng/mL)	2.275	1.229–4.210	<0.01	1.505	0.760–2.979	0.24
Preoperative CA199 (≥37 vs. <37 ng/mL)	1.001	0.982–1.021	0.91			
Operation method (open vs. laparoscopic)	1.168	0.0.685–1.992	0.57			
Type of surgery (APR + Hartmann's vs. anterior resection)	1.549	0.854–2.811	0.15			
Operation time (minutes)	1.002	0.998–1.006	0.36			
Blood loss (ml)	1.001	0.999–1.003	0.55			
Complication	1.446	0.818–2.554	0.20			
Tumor size (≥2 vs. <2 cm)	1.816	0.946–3.484	0.07			
ypT stage (ypT3-4 vs. ypT0-2)	1.611	0.874–2.971	0.13			
ypN stage (ypN2 vs. ypN1)	1.712	0.969–3.025	0.06			
ypTNM stage			0.11			
IIIA	Reference					
IIIB	1.449	0.747–2.812	0.27			
IIIC	2.674	1.075–6.654	0.03			
No. of lymph nodes harvested	1.025	0.985–1.065	0.22			
LND			<0.01			<0.01
LND1	Reference			Reference		
LND2	1.893	0.991–3.616	0.05	1.931	1.007–3.704	0.05
LND3	5.901	3.003–11.596	<0.01	6.035	3.058–11.909	<0.01
Tumor differentiation (poorly differentiated, others^a^ vs. Well moderately differentiated)	1.302	0.717–2.361	0.39			
Tumor regression grade			0.73			
0	Reference					
1	1.088	0.251–4.709	0.91			
2	1.167	0.280–4.864	0.83			
3	1.659	0.363–7.574	0.51			
Perineural invasion	2.091	1.088–4.017	0.03	1.686	0.854–3.327	0.13
Lymphovascular invasion	1.559	0.565–4.296	0.39			
CRM involvement	3.433	0.835–14.107	0.09			

*CEA, serum carcinoembryonic antigen; CA199, carbohydrate antigen 19-9; APR, abdominoperineal resection; LND, lymph node distribution; ^a^Included mucinous and signet ring cell carcinoma; CRM, circumferential resection margin*.

**Table 4:** Cox regression analysis of factors for 3-year distant metastasis-free survival in ypN+ rectal cancer patients after nCRT.

**Factors**	**Univariate analysis**			**Cox regression analysis**		
	**HR**	**95%CI**	***P***		**HR**	**95%CI**	***P***
Gender (male vs. female)	0.963	0.596-1.555	0.88				
Age (year)	1.000	0.981-1.019	0.96				
**ASA class**							
1	Reference		0.72				
2	1.074	0.599-1.925	0.81				
3	2.212	0.306-15.999	0.43				
Interval to surgery (weeks)	0.858	0.750-0.981	0.03		0.947	0.947-0.821	0.45
Preoperative chemotherapy regimen (fluorouracil + oxaliplatin vs. fluorouracil only)	1.446	0.911-2.295	0.12				
Distance from anal verge (≤ 5 vs. >5 cm)	0.854	0.539-1.355	0.50				
Preoperative CEA (≥5 vs. <5 ng/mL)	2.477	1.417-4.331	<0.01		1.791	0.980-3.271	0.06
Preoperative CA199 (≥37 vs. <37 ng/mL)	1.31	0.568-3.022	0.81				
Operation method (open vs. laparoscopic)	1.576	0.980-2.536	0.06				
Type of surgery (APR + Hartmann's vs. anterior resection)	1.370	0.776-2.418	0.28				
Operation time (minutes)	1.000	0.997-1.004	0.80				
Blood loss (ml)	1.001	0.999-1.003	0.27				
Complication	1.343	0.789-2.288	0.28				
Tumor size (<2 vs. ≥2 cm)	1.461	0.828-2.579	0.19				
ypT stage (ypT3-4 vs. ypT0-2)	2.184	1.198-3.980	0.01				
ypN stage (ypN2 vs. ypN1)	1.364	0.793-2.348	0.26				
ypTNM stage			0.06				
IIIA	Reference						
IIIB	1.993	1.041-3.812	0.04				
IIIC	2.782	1.119-6.919	0.03				
No. of lymph nodes harvested	0.993	0.957-1.030	0.70				
LND			<0.01				<0.01
LND1	Reference				Reference		
LND2	1.98	1.105-3.549	0.02		1.96	1.089-3.529	0.03
LND3	4.746	2.496-9.025	<0.01		3.885	1.940-7.777	<0.01
Tumor differentiation (poorly differentiated, others ^a^ vs. well moderately differentiated)	1.752	0.943-3.256	0.08				
Tumor regression grade			0.27				
0	Reference						
1	1.272	0.296-5.464	0.75				
2	1.512	0.365-6.264	0.57				
3	2.463	0.556-10.920	0.24				
Perineural invasion	1.752	0.943-3.256	0.08				
Lymphovascular invasion	1.87	0.754-4.640	0.18				
CRM involvement	3.014	0.737-12.327	0.13				

*CEA, serum carcinoembryonic antigen; CA199, carbohydrate antigen 19-9; APR, abdominoperineal resection; LND, lymph node distribution; ^a^Included mucinous and signet ring cell carcinoma; CRM, circumferential resection margin*.

### Prognostic Comparison of Special Distribution of Lymph Nodes

The 3-year DFS of the patients with LNDm, LNDp, and LNDmp were 66.8, 38.5, and 34.2%, respectively. There was no considerable difference between LNDp and LNDmp (*P* = 0.304). LRFS and DMFS showed similar results (Supplementary Figure 1).

## Discussion

The current study demonstrated the prognostic value of LND in patients with LARC after nCRT. In patients with positive lymph nodes, metastasis at the proximal lymph nodes is a sign of worse prognosis. LND showed better prognostic stratification than the ypN and ypTNM staging systems for local recurrence or distant metastasis.

Previous studies have shown that the prognostic significance of LND in colorectal cancer remains controversial (10–14). Several studies from Korea (10, 11) reported that LND is an independent prognostic factor in sigmoid and rectal cancer, while in right-sided and descending colon cancer, it is less meaningful than the pN category (13, 14). The current pN staging is based on the number of lymph node metastases, which requires the collection of sufficient lymph nodes. However, radiotherapy leads to shrinkage and fibrosis of both the primary lesion and regional lymph nodes, which causes a reduction in the number of both metastatic and harvested lymph nodes (21). Therefore, the prognostic value of pN staging in LARC after nCRT may decline accordingly (22). Other lymph node assessment methods, such as LND, are needed to compensate for the limitations of pN staging. Till date, there have been few studies on the prognostic significance of LND in rectal cancer after nCRT. Leibold et al. (18) reported that proximal lymph node metastasis is related to distant metastasis of rectal cancer after nCRT; however, the study included a relatively small sample (*n* = 121) and no specific prognostic information. In another study, LND could improve the prognostic value of the ypTNM staging system in patients with rectal cancer after nCRT, especially in terms of local recurrence. However, it did not divide the proximal lymph node into intermediate and main lymph nodes (16). Previous studies have confirmed that LND3 is an independent factor for the prognosis of rectal cancer, and the prognostic significance of LND2 is usually ignored. In our study, significant difference was found in prognosis between LND2 and other groups (LND1 vs. LND2, *P* = 0.013; LND2 vs. LND3, *P* = 0.03). In brief, LND can be used as an evaluation indicator supplementary to the ypTNM staging system. For patients with proximal lymph node metastasis with worse prognosis, individualized adjuvant treatment and more frequent postoperative monitoring may improve survival.

The prognostic model using LND is dependent on high ligation of the IMA and dissection of lymph nodes at the root of IMA. In the latest AJCC staging system, IMA lymph nodes are defined as regional lymph nodes. Several studies (11, 15) have reported that LND3 in rectal cancer indicates tumor progression. The prognosis of patients with LND3 is similar with that of patients with stage IV disease; thus, it could be considered as distant metastasis. Dissection of lymph nodes at the root of IMA seems to be a necessary option. However, it remains unclear whether high ligation of IMA and complete resection of IMA lymph nodes will benefit survival. Previous studies have demonstrated that high ligation of IMA lymph nodes can benefit survival, but this may be a result of staging shift effects (23). Recent studies have found no significant difference in the survival between high or low ligation of IMA. Besides, high ligation may lead to decreased anastomotic blood perfusion and autonomic nerve disorders (24, 25). Thus, the current evidence seems to favor low ligation of IMA. In patients undergoing primary surgery, IMA lymph node metastasis rates range from 0.3 to 8.6% (26). Sun et al. (15) reported that nCRT can reduce the number of IMA lymph node metastases (4.3 vs. 10.1%, *P* = 0.004). In our study, 13 patients (1.8%) had IMA lymph node involvement. If IMA lymph node is not dissected in these patients, R0 resection cannot be performed. The potential survival benefit may be significant when IMA lymph node metastasis is predicted. Therefore, further research is needed to confirm whether high or low ligation IMA affects survival of patients with rectal cancer after undergoing nCRT.

In addition, there were 13 patients (1.8%) with only metastasis at proximal lymph nodes (LNDp). LNDp in rectal cancer after nCRT was rarely reported. Lee et al. (16) demonstrated that LNDp was found in 2.8% patients with LARC following nCRT, and they showed better prognosis. The authors believe that the regression of mesorectal lymph nodes may represent a higher response to nCRT, leading to better local control. The incidence of LNDp is low; however, it should not be ignored. Its mechanism has not been clarified. It may involve lymphatic bypass, lymph node micrometastases, and defibrotic fibrosis following nCRT (27, 28). In our study, patients with LNDp seemed to exhibit better survival than those with LNDmp and worse survival than those with LNDm; however, this difference was not significant, which may be related to the small sample. Therefore, we classify the patients with LNDp into the LND2 or LND3 groups. In conclusion, a more optimized study design may help reveal the specific mechanisms and prognosis of LNDp.

We observed a higher incidence of postoperative complications in patients with proximal lymph node metastasis. This may be related to the increase in implementation of open surgery, APR, and Hartmann's surgery. At present, laparoscopic surgery and open surgery do not differ with regard to prognosis; however, the former can lead to a better short-term outcome. However, the surgeon at our center believes that patients with more advanced disease tend to be treated by open surgery. When bulky tumors, tumors invading adjacent organs, and serious violation in the main lymph nodes are found before or during the operation, open surgery is performed as it allows better visualization and operating space.

There are several limitations of this study. First, despite the large sample size in this center (*n* = 725), the number of LND3 cases is extremely low (*n* = 13). The application of nCRT decreased the number of lymph node metastases at the root of the IMA; however, it continued to result in extremely worse prognosis. Second, the prognosis of LNDp remains unclear. In this study, the number of cases of LNDp was low (*n* = 13), and we classified it under LND2 or LND3. Third, no comparison was performed with patients who have not received nCRT. Fourth, due to the retrospective design, this study has a selection bias.

As we have observed, patients with stage III rectal cancer after nCRT account for only 24.7% (179/725) of the total, which greatly limits our study sample. In addition, it is one-sided to evaluate prognosis only by locating the site of lymph node metastasis. To negate this limitation, the number of lymph node metastases can be combined with the location to build a better prognostic model. The follow-up results will be shown in future research from our center.

In this cohort of 725 patients with rectal cancer who received neoadjuvant therapy at a single comprehensive cancer center, proximal lymph node involvement was associated with a higher risk of recurrence. Therefore, it is necessary to adopt more effective treatment strategies to improve the prognosis of patients with LND2 and LND3.

## Data Availability Statement

The raw data supporting the conclusions of this article will be made available by the authors, without undue reservation.

## Ethics Statement

The studies involving human participants were reviewed and approved by the Ethics Committee of Fujian Medical University Union Hospital. The patients/participants provided their written informed consent to participate in this study.

## Author Contributions

BC: conception and design of the study. BC, YZ, YWa, YWu, and YP: acquisition of data. BC, XL, YY, and SL: analysis and interpretation of data. BC, XL, and YZ: drafting the article. XL, JZ, and GG: critical revision. All authors have approved the final manuscript and agree to be accountable for the content of the work.

## Funding

This work was supported by the Science Foundation of the Fujian Province (No. 2016J01602 and 2019J0105), Special Financial Foundation of Fujian Provincial (No. 2015-1297), Young and middle-aged backbone training project in the health system of Fujian province (2016-ZQN-26), the Startup Fund for Scientific Research, Fujian Medical University (2017XQ1029 and 2018QH2027), and the Professor Development Foundation of Fujian Medical University (No. JS11006).

## Conflict of Interest

The authors declare that the research was conducted in the absence of any commercial or financial relationships that could be construed as a potential conflict of interest.

## Publisher's Note

All claims expressed in this article are solely those of the authors and do not necessarily represent those of their affiliated organizations, or those of the publisher, the editors and the reviewers. Any product that may be evaluated in this article, or claim that may be made by its manufacturer, is not guaranteed or endorsed by the publisher.
