# Supplementary material for: Prognostic Value of the Distribution of Lymph Node Metastasis in Locally Advanced Rectal Cancer After Neoadjuvant Chemoradiotherapy
Source: Front Surg. 2021 Nov 17;8:749575. doi: 10.3389/fsurg.2021.749575 (PMC8635484; doi:10.3389/fsurg.2021.749575)
Supplement: Supplementary file 1 [file Data_Sheet_1.docx]

Supplementary Material

## Supplementary Figures

**
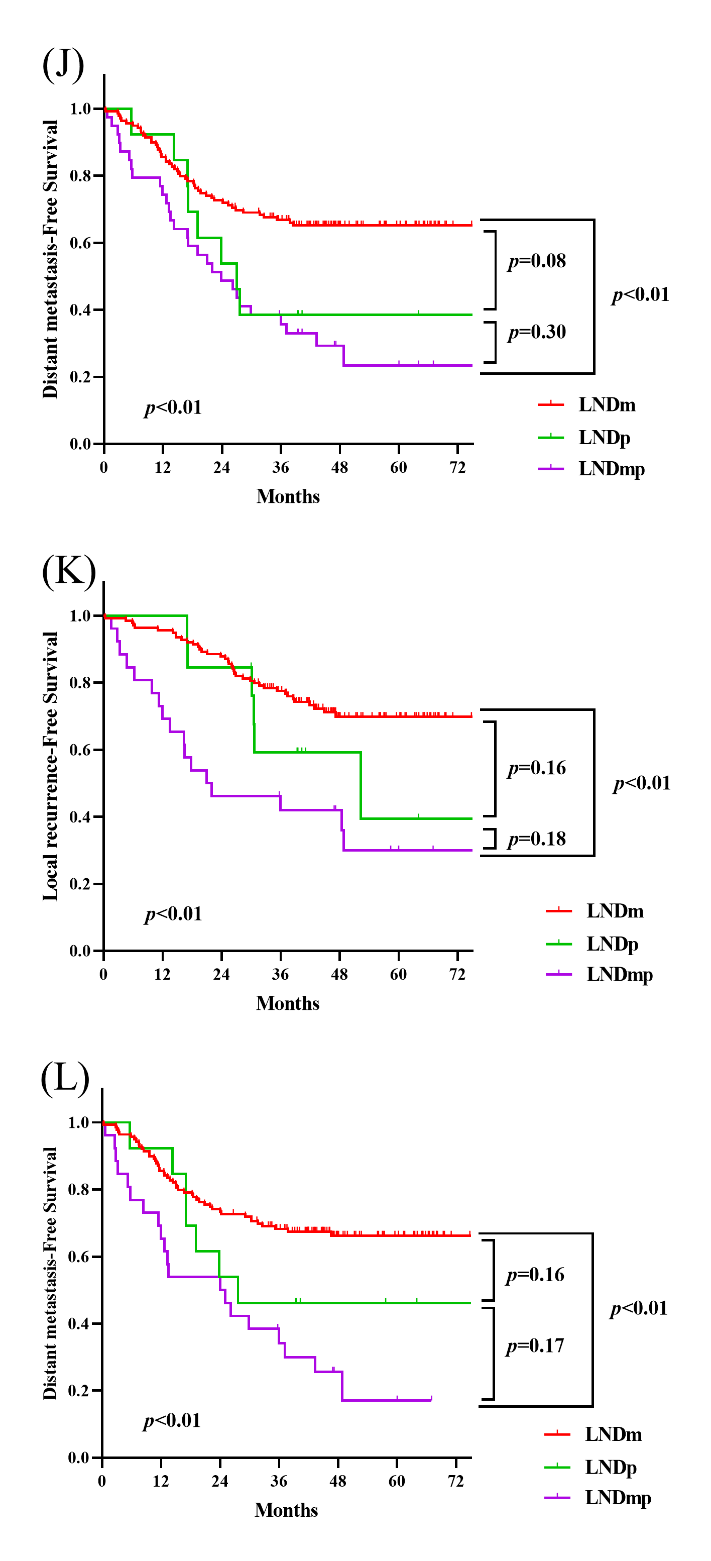
**

**Supplementary Figure 1.** Kaplan-Meier survival curve among LNDm, LNDp, and LNDmp (J, K, L). J, disease-free survival; K, local recurrence-free survival; L, distant metastasis-free survival
